# Fainting Fits and How to Cure Them

**Published:** 1871-02

**Authors:** 


Fainting Fits, and How to Cure Them.—Fainting or syncope is a partial or complete cessation of the action of the heart, a loss of consciousness, and a diminished or suspended respiration. The treatment should be :

1. Place the person in a horizontal position on the back. Do not bolster up the head, but leave it on a level with or a little below the body.

2. Secure fresh air for him at once, and, if possible, let it blow across his face, but if not, fan him. If he is in a close, crowded room, remove him immediately, but keep him in the horizontal position while doing so.

3. Loosen all clothing about the throat, chest, waist and abdomen.

4. Dismiss every person from the room, except such as can render the necessary assistance.

5. Sprinkle cold water upon the head and face. If that does not effect the desired result, pour a stream of cold water from a height of several feet upon the head. When the patient can swallow, a drink of cold water should be given.

6. Rub briskly and slap sharply the palms of the hands and soles of the feet, and, in obstinate cases, do the same to the whole surface of the body.

In most cases the horizontal position and pure air are sufficient to restore the patient. When caused by drinking a large quantity of cold water, a liberal amount of hot water should be given immediately. When a person feels the premonitory symptoms of fainting, as many do, he should at once place himself flat on his back. This alone will usually prevent the fit.—*Herald of Health.*

